# Choroidal thickness in patients with coronary artery disease

**DOI:** 10.1371/journal.pone.0175691

**Published:** 2017-06-20

**Authors:** Meleha Ahmad, Patrick A. Kaszubski, Lucy Cobbs, Harmony Reynolds, Roland Theodore Smith

**Affiliations:** 1 Department of Ophthalmology, New York University School of Medicine, New York, New York, United States of America; 2 Cardiovascular Clinical Research Center, Leon H. Charney Division of Cardiology, Department of Medicine, New York University School of Medicine, New York, New York, United States of America; Charite Universitatsmedizin Berlin, GERMANY

## Abstract

**Purpose:**

To evaluate choroidal thickness (CTh) in patients with coronary artery disease (CAD) compared to healthy controls.

**Design:**

Cross-sectional.

**Methods:**

Setting: Ambulatory clinic of a large city hospital. Patient population: Thirty-four patients had documented CAD, defined as history of >50% obstruction in at least one coronary artery on cardiac catheterization, positive stress test, ST elevation myocardial infarction, or revascularization procedure. Twenty-eight age-matched controls had no self-reported history of CAD or diabetes. Patients with high myopia, dense cataracts, and retinal disease were excluded. Observation procedures: Enhanced depth imaging optical coherence tomography and questionnaire regarding medical and ocular history. Main outcome measures: Subfoveal CTh and CTh 2000 μm superior, inferior, nasal, and temporal to the fovea in the left eye, measured by 2 readers.

**Results:**

CTh was significantly lower in patients with CAD compared to controls at the subfoveal location (252 vs. 303 μm, *P* = 0.002) and at all 4 cardinal macular locations. The mean difference in CTh between the 2 groups ranged from 46 to 75 μm and was greatest in the inferior location. Within the CAD group, CTh was significantly lower temporally (*P* = 0.007) and nasally (*P*<0.001) than subfoveally, consistent with the pattern observed in controls. On multivariate analysis, CAD was negatively associated with subfoveal CTh (*P* = 0.006) after controlling for diabetes, hypertension, and hypercholesterolemia.

**Conclusions and relevance:**

Patients with CAD have a thinner macular choroid than controls, with preservation of the normal spatial CTh pattern. Decreased CTh might predispose patients with CAD to high-risk phenotypes of age-related macular degeneration such as reticular pseudodrusen and could serve as a potential biomarker of disease in CAD.

## Introduction

The choroid supplies blood to the outer one-third of the neuroretina and the retinal pigment epithelium (RPE) and represents the sole provider of oxygen and nutrients to the avascular fovea. Despite its function in maintaining the retina, details of the choroidal circulation remained largely unknown due to poor resolution and reproducibility of previous choroidal imaging techniques, such as indocyanine green angiography [1] and ultrasound [2]. Imaging of the choroid was dramatically improved with the development of spectral domain optical coherence tomography (SD-OCT) and was further augmented with the advent of enhanced depth imaging SD-OCT (EDI SD-OCT) by Spaide and colleagues in 2008 [3]. Developing techniques, such as swept source optical coherence tomography (SS-OCT) and OCT angiography [4], have allowed segments of the choroid to be visualized down to nearly the capillary level, opening up a new world of research in this previously underexplored ocular tissue.

The imaging techniques described above have allowed for the study of the choroid in both a qualitative and a quantitative manner. In particular, much attention has been paid to choroidal thickness (CTh), a structural parameter that is typically defined as the distance between the outer border of the hyperreflective RPE and the hyperreflective inner border of the sclera on SD-OCT [3]. CTh can be easily measured using inbuilt calipers on OCT imaging software, making it accessible in research and clinical settings. While the true correlation between CTh and *in vivo* choroidal function, such as choroidal blood flow, remains uncertain [5], CTh is the closest objective marker of choroidal health available with present imaging techniques, making it a topic of great interest in outer retinal health.

CTh has been found to decrease significantly with age [6] and to vary with numerous systemic and ocular diseases [7]. The healthy choroid typically measures 250–400 μm subfoveally [8], with a decrease in thickness in the temporal and nasal directions [8,9]. The relationship between the choroid and cardiovascular disease (CVD) is of particular interest due to its possible use as a biomarker of CVD and in identifying patient cohorts at increased risk for outer retinal disease. Because the choroid is a highly vascular end organ with the greatest blood flow per mm^3^ in the body [10], it might be susceptible to arteriosclerotic processes common in other end organs. However, studies of CTh in patients with systemic diseases have shown variable results. Severe hypertensive retinopathy with serous retinal detachments has been associated with hypertensive choroidopathy and choroidal thickening [11]. Uncomplicated hypercholesterolemia without other vascular disease has been associated with choroidal thickening [12], whereas cigarette smoking [13,14], ocular ischemic syndrome [15], chronic heart failure [16], and systemic essential hypertension [17,18] have been linked to a thinner choroid. Carotid artery stenosis [19,20] and diabetes without diabetic retinopathy [21,22] have shown contradictory associations with CTh. In this study, we compared macular CTh in 34 patients with CAD to macular CTh in 28 healthy controls. Decreased CTh in patients with CAD would support a connection between cardiac disease and outer retinal diseases such as age-related macular degeneration (AMD).

## Methods

### Subject recruitment and imaging

Subjects and controls were recruited between January 2014 and September 2015 from the outpatient cardiac and primary care clinics of a large city hospital. New York University School of Medicine Institutional Review Board approval was obtained (Federalwide Assurance #00004952). Inclusion criteria for patients with CAD were as follows: clinically documented history of cardiac catheterization demonstrating greater than 50% obstruction in at least one coronary artery, positive stress test, ST segment elevation myocardial infarction (MI), or revascularization procedure (stent or coronary artery bypass graft). Controls included patients without a documented or self-reported history of CAD (including procedures/conditions listed above) or CAD-equivalent conditions, including peripheral artery disease, history of stroke, or diabetes. Patients with high myopia (> 6D), AMD, advanced cataracts, or a history of retinal vascular disease, retinal dystrophy, retinal surgery, or laser photocoagulation were excluded from both the CAD and control groups. Patients with CAD were age-matched to controls.

After obtaining written informed consent, all subjects completed a detailed questionnaire regarding ocular and medical history, with a focus on CVD. Subjects then underwent near infrared and EDI SD-OCT imaging of both eyes using the Heidelberg Spectralis HRA+OCT (Heidelberg Engineering, Inc., Franklin, MA, USA) with eye-tracking ability. Macular volume scans consisted of 16 horizontal lines, each line an average of 9 B-scans, in a 15° by 20° rectangular pattern. Images with quality < 20 dB were excluded.

### Image analysis

**Fig 1:**
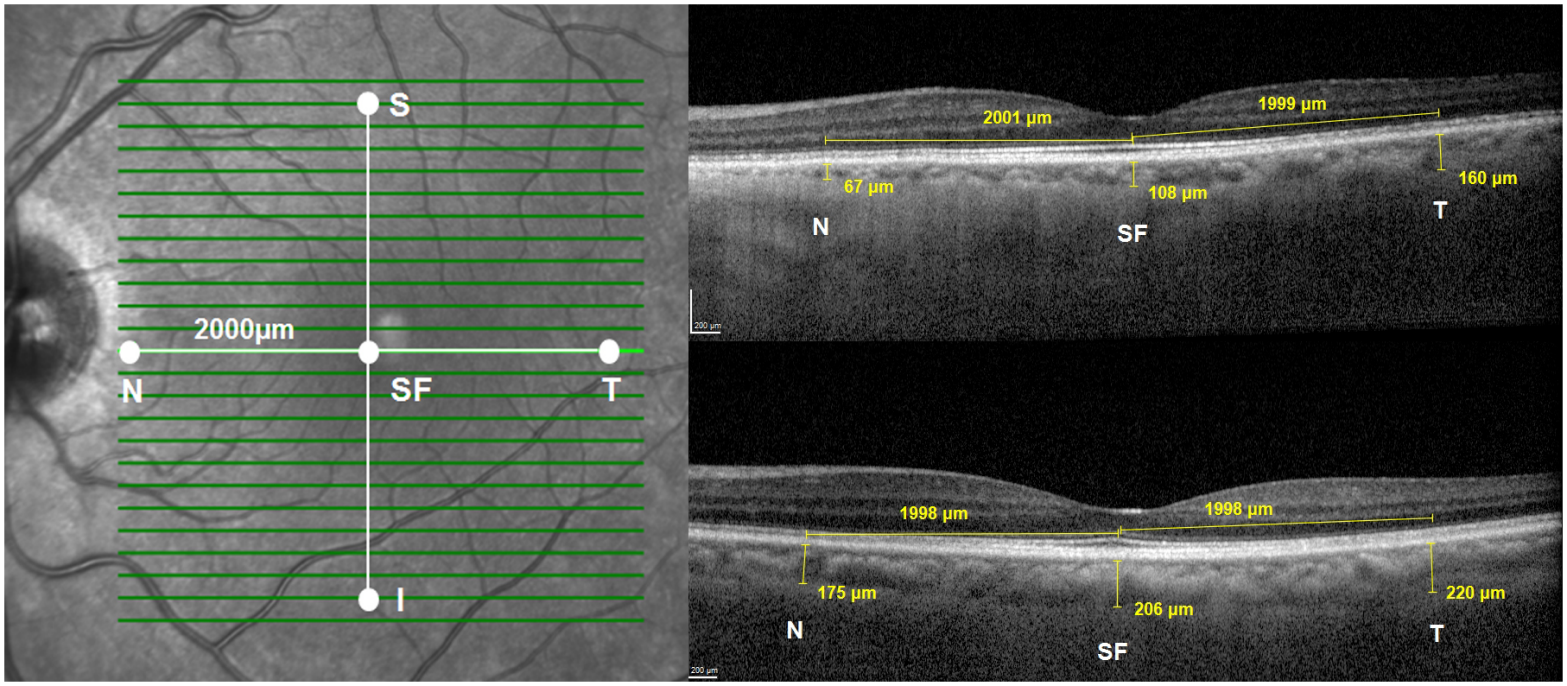
Choroidal thickness measurements. **Left.** Measurements were taken at five locations: subfoveal (SF), nasal (N), temporal (T), superior (S) and inferior (I) **Right.** Sample EDI SD-OCT foveal slice images showing choroidal thickness measurements at SF, N and T locations in a 72 year old woman with CAD (top) and healthy 70 year old woman (bottom). EDI SD-OCT, enhanced depth imaging spectral domain optical coherence tomography; CAD, coronary artery disease.

CTh was measured by 2 trained independent graders (MA and LC) using a built-in ruler tool in the Heidelberg Eye Explorer software (Fig 1). CTh measurements were averaged between the two readers. The left eye of each subject was selected for analysis. Readers were blinded to CAD status at the time of image reading. CTh was defined as the distance between the outer border of the hyperreflective RPE and the hyperreflective inner surface of the sclera. CTh was measured below the fovea, which was defined as the lowest point of the retina visible on macular SD-OCT slices, and 2000 μm away from the fovea in 4 cardinal macular regions: superior, inferior, temporal, and nasal. The superior and inferior points corresponded to 8 slices above and 8 slices below the foveal slice; the temporal and nasal points were identified using the ruler tool at the foveal slice (Fig 1). In cases of poor image slice quality, non-centered scans, or scans in which the sclerochoroidal border was not visible, no measurement was taken at the point in question.

### Statistical analysis

Statistical analysis was performed using Microscoft Excel (Microsoft Corp., Redmond, WA, USA) and SPSS 22.0 (IBM Corp., Armonk, NY, USA). For all tests, a p-value less than 0.05 was considered statistically significant. An inter-observer correlation coefficient was calculated for CTh measurements by two readers. In addition, measurements were repeated by one of the readers (MA) for a subset of images to calculate an intra-observer correlation. CTh was compared pointwise between patients with CAD and controls, and the macular pattern of CTh was also compared between patients with CAD and controls. Multivariate linear regression was conducted to evaluate relative effects of potential confounders on CTh.

## Results

**Fig 2:**
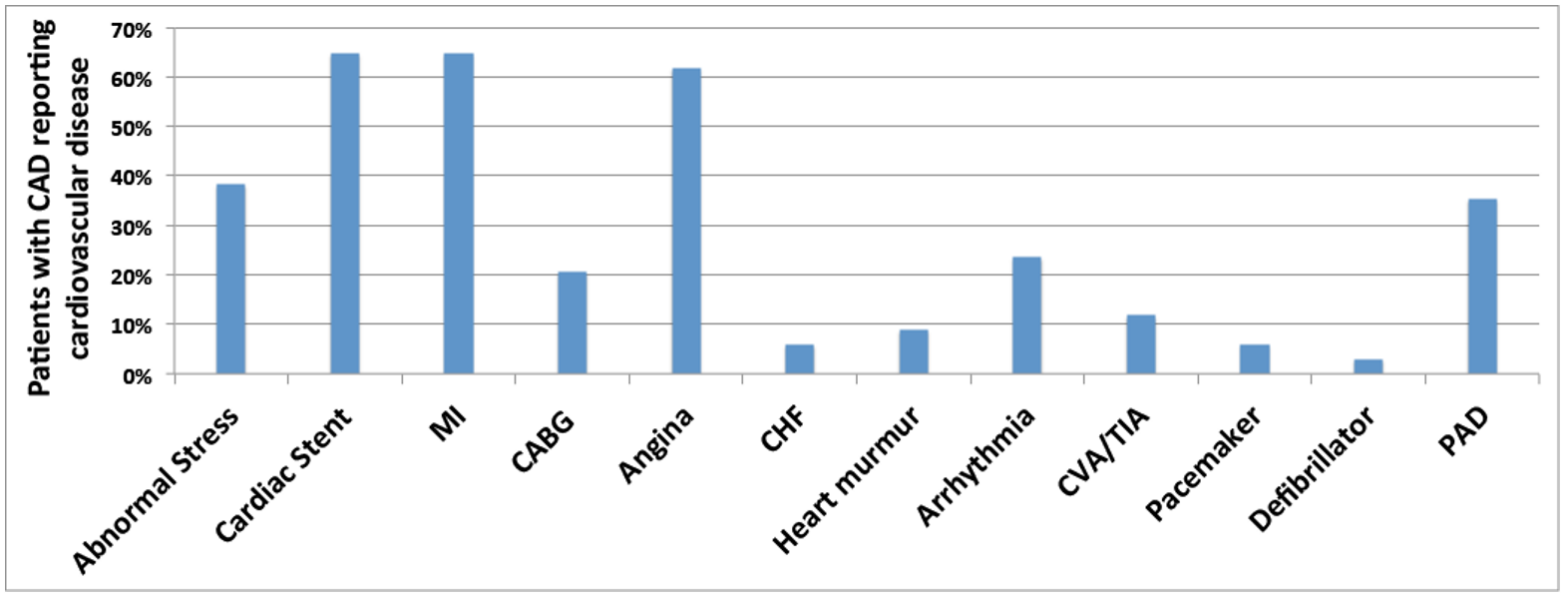
Cardiovascular history in patients with coronary artery disease. The majority of patients had a history of cardiac stent, angina or MI. Not shown: 0% of patients reported having a cardiac transplant or aneurysm. CAD, coronary artery disease; MI, myocardial infarction; CABG, coronary artery bypass graft; CHF, congestive heart failure; CVA, cerebrovascular accident; TIA, transient ischemic attack; PAD, peripheral artery disease.

Complete data was collected for 34 patients with documented CAD and 28 healthy controls. The mean age was 60.9 ± 6.8 years (range 45–76 years) for patients with CAD and 59.9 ± 5.2 years (range 51–71 years) for controls (*P* = 0.51; Mean Difference: 1 year, 95% confidence interval (CI): -4.1, 2.18). Characteristics of the study and control groups are shown in Table 1. Baseline demographics, including gender and ethnicity, were comparable between the 2 groups. Patients with CAD were more likely to have hypertension, hyperlipidemia, and diabetes compared to controls. Prevalences of various cardiovascular diagnoses in the CAD group are shown in Fig 2. Nearly 70% of the CAD population had suffered an MI, while the remaining had other evidence of CAD, such as a history of an abnormal stress test or a history of previous positive cardiac catheterization.

**Table 1:** Baseline characteristics of coronary artery disease study population compared with control population.

	CAD	Control	*P-*value
Number of patients	34	28	
Age, years (mean ± SD)	61.1 ± 6.8	60.1 ± 5.3	*P* = 0.5
Gender (n, % female)	15, 44.1%	17, 60.8%	*P* = 0.2
Ethnicity			
n, % White	7, 20.6%	6, 21.4%	*P* = 0.2
n, % Black	3, 8.8%	9, 32.1%	
n, % Hispanic	14, 41.1%	7, 25.0%	
n, % Asian	10, 29.4%	4, 14.3%	
Hypertension (n, %)	29, 85.3%	6, 21.4%	*P*<0.0001
Hypercholesterolemia (n, %)	28, 82.3%	9, 32.2%	*P*<0.0001
Diabetes (n, %)	16, 47.1%	0, 0.0%	*P*<0.0001
Kidney disease (n, %)	6, 17.6%	2, 7.1%	*P* = 0.1
Ever smoker (n, %)	21, 61.7%	16, 57.0%	*P* = 0.8

CAD, coronary artery disease; SD, standard deviation.

**Fig 3:**
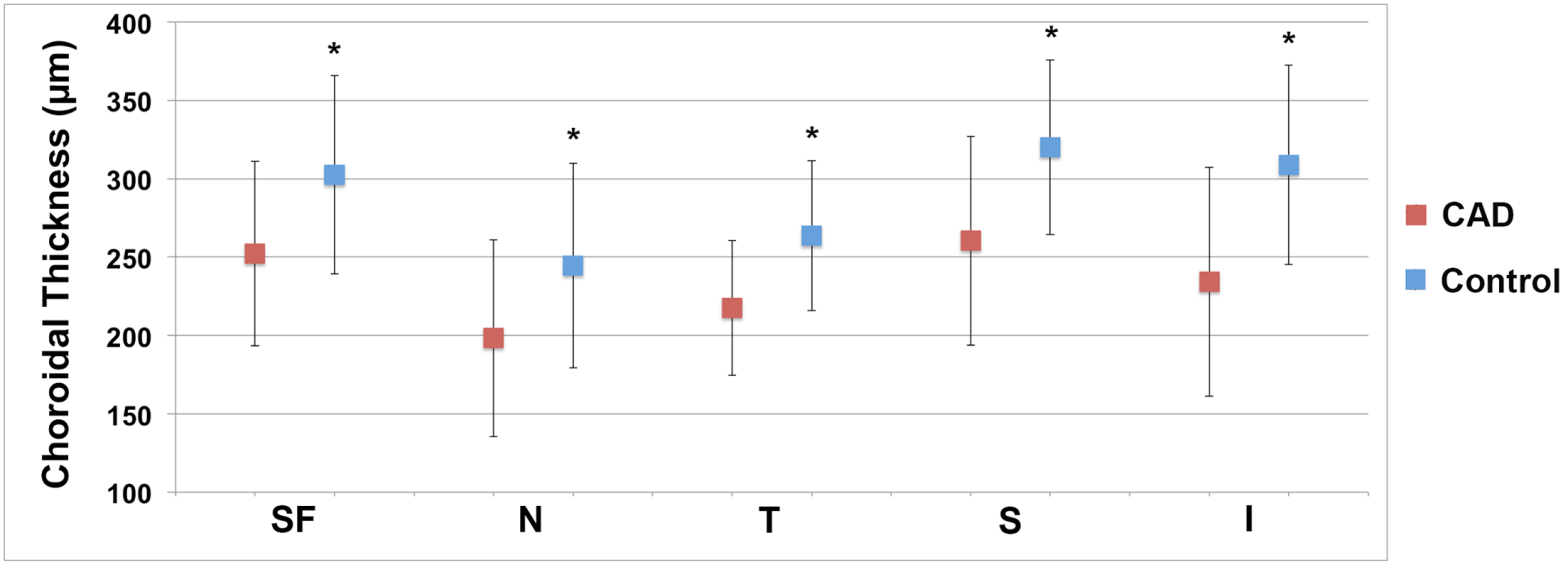
Subfoveal choroidal thickness in coronary artery disease compared to controls. Mean choroidal thickness at all 5 macular locations was lower in patients with coronary artery disease (red squares) than controls (blue squares). Average choroidal thickness was greatest at the subfoveal, inferior, and superior locations in both groups. Error bars represent standard deviation. SF, subfoveal; N, nasal; T, temporal; I, inferior.

The inter-observer correlation coefficient was 0.79 for the 2 CTh readers. The intra-observer correlation coefficient was 0.95 for CTh reader MA. A significantly thinner choroid was observed at the fovea of eyes in the CAD group compared to controls (252 vs. 303 μm, *P* = 0.002; 95% CI: 30.2, 88.8). In addition, CTh in the CAD group was thinner at all 4 cardinal macular points compared to controls (Table 2 and Fig 3). Differences in CTh between the CAD and control populations varied at each macular point from 46 to 75 μm, depending on the location, with the inferior location showing the greatest difference and the nasal and temporal locations showing the least difference.

**Table 2:** Subfoveal choroidal thickness and choroidal thickness at 4 cardinal macular locations in the left eye. All 5 locations showed significantly decreased CTh in patients with coronary artery disease compared with controls.

	CAD (N = 34)		Control (N = 28)					
	Mean (SD)	Min/Max	Mean (SD)	Min/Max	Mean CTh Difference	95% CI	*P*-value	*P*-value adjusted*
SF CTh (μm)	252 (58.8)	124/341	303 (63.2)	139/416	50.2	19.2, 81.3	*P* = 0.002	*P* = 0.006
N CTh (μm)	198 (62.8)	48.5/ 314	245 (65.3)	86.0/369	46.4	14.5, 80.6	*P* = 0.006	*P* = 0.04
T CTh (μm)	218 (43.0)	134/329	264 (47.8)	134/342	46.2	22.3, 70.2	*P*<0.001	*P* = 0.003
S CTh (μm)	261 (66.6)	117/357	320 (55.7)	172/414	59.7	26.9, 92.5	*P*<0.001	*P* = 0.003
I CTh (μm)	234 (73.1)	106/370	309 (63.6)	171/437	74.5	38.5, 110.6	*P* = 0.007	*P* = 0.005

* Adjusted for diabetes, hypertension, and hypercholesterolemia.

CAD, coronary artery disease; SD, standard deviation; CI: confidence interval; CTh, choroidal thickness; SF, subfoveal; N, nasal; T, temporal; S, superior; I, inferior.

CTh at the 4 cardinal macular locations was compared to subfoveal CTh for subjects with CAD and controls to assess the pattern of CTh. In subjects with CAD, there was no significant difference between subfoveal CTh and CTh at the superior or inferior locations; in contrast, the choroid was significantly thinner at the nasal and temporal locations when compared to the fovea (Table 3A). A similar pattern was observed in the control group, with the superior, inferior, and subfoveal locations having similar CTh and the nasal and temporal locations being thinner than the subfoveal CTh (Table 3B).

**Table 3:** Pattern of choroidal thickness in eyes of patients with coronary artery disease, showing significantly decreased CTh in the nasal and temporal locations compared with the subfoveal location, consistent with the pattern observed in control eyes.

	CAD (N = 34)		CONTROL (N = 28)	
	Average CTh Difference (μm)	P-value	Average CTh Difference (μm)	P-value
SF to S	8.2	*P* = 0.6	17.7	*P* = 0.3
SF to I	17.9	*P* = 0.3	18.0	*P* = 0.7
SF to N	54.0	*P*<0.001	57.8	*P* = 0.001
SF to T	34.8	*P* = 0.007	38.8	*P* = 0.02

CAD, coronary artery disease; CTh, choroidal thickness; SF, subfoveal; S, superior; I, inferior; N, nasal; T, temporal.

Multivariate linear regression was conducted to evaluate the effect of diabetes, hypertension, and hypercholesterolemia on subfoveal CTh. A strong negative association between CAD and CTh remained even after controlling for all 3 potential confounders (*P* = 0.006).

## Discussion

CAD is the leading cause of death worldwide in both men and women [23], accounting for 1 in every 4 deaths in the United States [24] and 31% of deaths worldwide [25]. Despite the use of multiple clinical markers and risk factors for CAD, there remains a continued interest in finding new clinical and examination tools to better assist in risk stratification for CAD. This is of particular importance in women, for whom typical risk stratification tools, such as the Framingham Risk Score, often fail to detect underlying cardiac disease [26], possibly due to the preponderance of atypical symptoms and coronary microvascular disease [27,28].

The use of ocular examination as a method of CAD risk stratification has been proposed due to its unique ability to view the vasculature of the posterior segment *in vivo* and in a non-invasive manner, thus providing a snapshot of vascular health. Until now, much focus has been on investigating the connection between *retinal* vasculature changes and CVD, likely due to the ease of viewing these vessels on clinical examination. There is a known connection between retinal arteriolar narrowing and cardiovascular events, at least in some subpopulations [29]. However, the use of retinal vasculature as a biomarker for CAD has been problematic due to the difficulty in quantifying retinal vascular findings in a standardized way [29]. The ability to easily visualize the choroid clinically using EDI SD-OCT provides new opportunities for research into both quantitative risk stratification in CAD using CTh and improved understanding of outer retinal health in CAD patients.

The choroid is typically described as having 5 layers: Bruch’s membrane, the choriocapillaris, Haller’s and Sattler’s vascular layers, and the suprachoroidea, or suprachoroidal space (a 10–15 μm layer of giant melanocytes interspersed between flattened processes of fibroblastic cells) [30,31]. The choriocapillaris is a network of fenestrated capillaries 20–40 μm in diameter arising from medium-sized arteries in Sattler’s layer and larger arteries in Haller’s layer [10]. As shown by Hayreh, using *in vivo* fluorescein angiography studies, the choroid is arranged in a lobular pattern, with each end artery supplying a single segment and no anastomoses between these segments [32]. With the advent of EDI SD-OCT, it is now quick and easy to visualize the choroid, which is the major blood supply of the outer neuroretina and RPE, and measure CTh.

Our major finding was a strong, independent negative association between history of CAD and CTh. CTh is known to be affected by a variety of systemic and ocular factors, of which age and axial length are 2 major ones [33]. Gender has also been associated with CTh differences, with most studies showing that men have greater CTh than women, likely due to hormonal factors and sympathetic tone [7,34]. Our CAD and control groups showed no significant difference in age or gender distribution, reducing the likelihood that these factors accounted for the differences in CTh that we observed. The seemingly obvious connection between the vascular components of the choroid and other vascular beds of the body has produced a number of studies on the relationship between CTh and various cardiovascular diseases and risk factors. A single study of 56 patients with congestive heart failure showed lower subfoveal CTh compared to age- and gender-matched controls [16]. Although hypertensive retinopathy has been associated with increased CTh [11], correlations between CTh and systemic hypertension in healthy retinas have been inconsistent, with one study showing significantly thinner CTh compared with healthy controls [17] and another showing no significant association [18]. Similarly, internal carotid artery stenosis has been variably associated with CTh, with one study showing a positive correlation between extent of stenosis and CTh [19] and another showing an inverse relationship [20]. A study by Agladioglu et al. noted an inverse relationship between internal carotid artery diameter and CTh; however, this finding was in healthy patients without stenosis [35]. A single study evaluated the relationship between hypercholesterolemia and CTh, showing CTh to be significantly higher in patients with increased total cholesterol compared to controls; however, all cases of hypercholesterolemia were treated [12]. In our CAD group, subfoveal CTh was significantly lower than that of normal controls, even after correction for the presence of hypertension, hypercholesterolemia, and diabetes.

The possible physiologic basis for a relationship between CTh and CAD is intriguing. The term CAD generally refers to the atherosclerotic disease of medium to large vessels, and choroidal vessel diameter is more on par with the coronary microvessels than the large-diameter coronary vessels [29, 36]. Microvascular coronary disease occurs when there is demonstrable coronary ischemia in the absence of the angiographically obstructive atherosclerosis seen in our CAD group. Study of the contribution of the coronary microvasculature to pathogenesis and events in patients with CAD has been limited by the challenge of observing the coronary microvasculature, which typically requires myocardial biopsy. Techniques such as myocardial contrast echocardiography may allow inference into microvascular function based on flow parameters, but this inference is not straightforward. Further studies will be required to parse out the contribution of coronary microvascular disease to the connection between CAD and decreased CTh.

Our finding of significantly lower CTh in patients with CAD has possible implications for retinal disease in patients with CAD. Both the photoreceptors and the entire fovea are highly dependent on the choroid for function, with over 90% of oxygen provided to the photoreceptors coming from the choroidal circulation [37]. A large number of studies have investigated the connection between CAD and AMD, producing mixed results. In a study by Duan et al., patients with choroidal neovascularization were 26% more likely to develop MI compared with controls after adjusting for age, gender, race, and hypertension at baseline [38]. Other studies have found similar relationships between early and late AMD and CVD and its risk factors [39–42]. However, conflicting studies have shown no relationship [43], or even an inverse relationship, between CVD and AMD [44–47]. Recent studies have suggested that reticular macular disease, a high risk sub-phenotype of AMD consisting of reticular pseudodrusen and decreased CTh, may have an even stronger correlation with CAD than does typical AMD [48,49]. Decreased CTh in the absence of retinal abnormalities in CAD may represent the precursor to reticular macular disease.

CTh is known to be thickest at the subfoveal region, with thinning occurring in the nasal and temporal directions [8,9]. Some lesions are known to differentially affect specific regions of the retina, such a reticular pseudodrusen which are most often found in the superior macula [48]. Reticular pseudodrusen also has a newly emerging association with CAD, and for this reason, we were particularly interested in understanding the topographical pattern of decreased CTh observed in CAD patients compared with controls. This pattern was replicated in the control population of our study but, importantly, also in the CAD population.

Our study has a number of limitations. The study groups were relatively small. Although patients with high myopia were excluded from the study groups, we did not collect quantitative axial length data and therefore cannot account for variations in CTh due to myopia of less than 6D, and thus cannot rule out any smaller effects of axial length on CTh. We did not have information on carotid artery stenosis for our CAD or control groups. In addition, because we recruited patients with CAD during the afternoon clinic and control patients during both the morning and afternoon clinic, we were unable to control for diurnal variation in CTh, which is thought to be 20–30 μm from morning to evening [50–52]. However, on further analysis of our imaging timings, the average difference between time of imaging of CAD compared to controls was approximately 2 hours, which would equate to a 5 μm or less difference between the 2 groups.

Strengths of the study include well-characterized, prospectively recruited subjects with CAD from cardiology clinics and carefully selected, age-matched controls. Poor-quality imaging data was excluded. The differences in CTh between the groups were highly significant, and these differences were found at 5 measurement points in the macula.

In conclusion, we evaluated CTh in a CAD group compared to age-matched controls, finding an independent, negative association between CTh and CAD. These findings suggest that CTh may serve as an important disease marker in CAD, providing important information on both systemic cardiovascular health and susceptibility to diseases of the outer retina and RPE. Our findings warrant future research on the connection between the choroid and other vital vascular systems of the body. Further studies may employ SS-OCT imaging in patients with CAD to better understand which choroidal layers are contributing to the decreased CTh we observed.

## Supporting information

S1 Table Choroidal thickness raw data. Choroidal thickness values at five cardinal macular locations for cases and controls prior to data analysis.
(XLSX)
